# Effect of brewing conditions using a single‐serve coffee maker on black tea (Lapsang Souchong) quality

**DOI:** 10.1002/fsn3.1735

**Published:** 2020-07-08

**Authors:** Chunhua Ma, Yen‐Con Hung

**Affiliations:** ^1^ College of Tea and Food Science Wuyi University Wuyishan City China; ^2^ Department of Food Science and Technology University of Georgia Griffin GA USA

**Keywords:** brewing, coffee maker, compounds, tea

## Abstract

Black tea powder was used to make tea infusion using a Keuring coffee maker. Effect of tea particle size (0.30 or 0.60 mm), water volume for brewing (118, 177, or 236 ml), and the amount of tea powder (1.0, 1.5, or 2.0 g) on tea infusion quality were evaluated. The concentration of four chemical compounds (soluble sugars, total amino acids, polyphenols, and caffeine) in tea infusion was measured. In general, the concentration of the four compounds in tea infusion increased with the amount of tea powder used and decreased with increasing water volume and tea particle size. Using 2.0 g of 0.30 mm tea powder and 118 ml of water per brew, the concentration of the above four components was 412.3, 251.6, 208.9, and 205.3 μg/ml, respectively, and higher than tea infusion prepared by traditional method (312.4, 204.1, 211.6, and 175.9 μg/ml). The highest extraction efficiency (mg/cup.g) for the four chemical components per unit weight of tea powder was at using 1.0 g of 0.30 mm tea powder with 236 ml water for soluble sugars (32.3 μg/ml) and caffeine (16.3 μg/ml), at using 1.5 g tea powders for polyphenols (22.3 μg/ml) and at using 2.0 g tea powders for amino acid (13.6 μg/ml). This study suggested using a single‐serve coffee maker can be a convenient and effective way to prepare the tea infusion with high quality.

## Graphical abstract

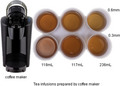

## 1 INTRODUCTION

Tea (*Camellia sinensis*) is one of the most consumed functional beverages in the world (Ho, Zheng, & Li, 2015). According to the degree of fermentation, tea is classified into three major categories: unfermented green tea, partially fermented Oolong tea, and fully fermented black tea, and they account for about 24%, 1%, and 75% of the total world tea production, respectively (Wang et al., 2008). Black tea is commonly consumed in the west, whereas green tea is especially popular in Asia. People like tea not only for its aroma but also for its health benefit, including strong antioxidant properties (Sharma et al., 2017), anticancer activity (Krstic, Stojadinovic, Smiljanic, Stanic‐Vucinic, & Velickovic, 2015; Ni et al., 2017), inhibition of inflammation, and protective effects against diabetes, hyperlipidemia, and obesity (Li, Lo, Pan, Lai, & Ho, 2013; Pan, Tung, Yang, Li, & Ho, 2016).

Most of tea is consumed as a tea drink, and the quality of tea can be affected by many factors, for example, tea processing conditions and brewing methods. There are many variations in brewing techniques which can impact both flavor and extraction efficiency of chemical compounds (Dos, Ayhan, & Sumnu, 2005; Lin, Yang, Hsieh, Liu, & Mau, 2014; Peterson et al., 2004). The most popular method of brewing is soaking dried tea leaves with hot water and has been adopted throughout the world (Gao et al., 2019). However, the pace of modern life is increasing with the advancement of technologies and fast‐paced living style making people now have little time to enjoy drinking tea that is prepared by traditional brewing. The coffee beverage industry was also facing this similar challenge, and one of the solutions was the development of a single‐serve coffee maker. Consumers can now enjoy a fresh cup of coffee anytime without brewing a large pot of coffee. Consumers also can change the coffee bean variety and concentration between each cup.

One convenient form of making tea drink is using instant tea powders like instant coffee powders. Instant tea is presently manufactured by drying off tea extract to form instant tea powders (Ye, Zhang, Sun, Chen, & Fang, 2014). While it is convenient to consume instant tea, manufacturing instant tea powders from tea extraction by drying consumed a lot of energy. During drying, some beneficial nutrients and aroma compounds may have also lost. Previous research on tea in the literature was mostly focused on brewing conditions, such as infusion time, water composition (Franks, Lawrence, Abbaspourrad, & Dando, 2019), and tea varieties on the quality like antioxidant capacity of infusions (Sharpe, Hua, Schuckers, Andreescu, & Bradley, 2016). Very little research was focused on the effect of particle size of tea leaves during extraction and extraction efficiency during one single brewing. The purpose of this research was to study the influence of amount and particle size of black tea on the quality of tea infusions brewed using a single‐serve coffee maker with different amount of water. The main chemical components in tea infusions measured were tea polyphenols, caffeine, soluble sugars, and total amino acids. In addition, chemical components in tea infusion brewed by a traditional method were also measured and compared with the tea infusion prepared by a single‐serve coffee maker.

## 2 MATERIALS AND METHODS

### 2.1 Tea infusion preparation

Tea used in the present study was black tea (lapsang souchong), produced in Fujian Province, China. Tea leaves were ground in a mixer (Elgi Ultra) by pulse grinding, and ground tea particles were sieved through 2 sieves at screen opening of 0.30 and 0.60 mm to obtain tea powders for the study. A single‐serve coffee maker was used to brew tea (K575, Keuring). Tea infusion was made according to the coffee maker instructions by placing 1.0, 1.5, or 2.0 g of tea powder into a reusable cup and then selecting different water volumes (118, 177, or 236 ml, equivalent to 4, 6, or 8 ounces). And brewing time was fixed by the coffee maker used and was 40 s for each brew. Tea quality was measured when tea infusions were cooled to room temperature (23°C). A traditional brewing (Kongfu) method was also used to prepare tea infusions by adding 5.0 g of tea leaves into a porcelain bowl and then pour boiled water over the leaves until the porcelain bowl is full (110 ml). After 30 s, tea infusion was then pull out from the porcelain bowl and cooled to room temperature (23°C) before further analysis. This tea brewing procedure was repeated for 6 more times with the soaking time increased gradually at the rate of about 10 s for each additional brew, from 30 s during the initial brew to 90 s for the last brew.

### 2.2 Determination of soluble sugars

Soluble sugar was determined according to the method of Liang, Syu, Lee, and Mau (2009). One ml of tea infusions was added to individual test tubes, and 4.0 ml of anthrone reagent was added to each test tube. The whole mixture was shaken thoroughly using a vortex mixer (IKA Vortex2) and then heated for 15 min at 100°C using a water bath (IKA RCT Basic). At the end of the heating treatment, test tubes were rapidly cooled down with running tap water, and then, the absorbance at 620 nm was measured using the same spectrophotometer described above.

### 2.3 Determination of tea polyphenols

Contents of polyphenols in tea infusions were determined by a spectrophotometric method (ISO 14502‐1, 2005). One ml of tea infusion was transferred into a 10‐ml graduated test tube, and then, 5.0 ml of 10‐fold Folin–Ciocalteu phenol dilution was added into each tube, mixed with tea infusion evenly. Within 8 min after the addition of the Folin–Ciocalteu phenol reagent, 4.0 ml of sodium carbonate solution was added into each tube and then maintained at 25°C for 60 min. The absorbance at 765 nm was measured using a 10‐mm path length cuvette in a spectrophotometer (Orino aquamate 8000; Thermo scientific).

### 2.4 Determination of caffeine

Caffeine was determined according to the national standard of China (BG/T 8312‐2013). Tea infusions (1.0 ml) were put into a 50 ml volumetric flask, followed by 1.0 ml of 0.01 mol/L hydrochloric acid, saturated lead subacetate solution (0.2 ml), and water to a constant volume before filtered by a filter paper (Waterman No. 1). The absorbance of the filtrate in a 10 mm quartz cell at 274 nm was recorded using the same spectrophotometer described before.

### 2.5 Determination of the total amino acids

The method for determination of amino acids in the tea infusion was based on Chinese national standard (BG/T 8314‐2013). One ml of tea solution, 0.5 ml of phosphate buffer solution, and 0.5 ml of ninhydrin solution were placed in a 10 ml test tube, and the tube was heated in a boiling water bath for 10 min. The tube was cooled to room temperature, and the absorbance of the solution at 570 nm was obtained.

### 2.6 Color measurement

The color of tea infusion was measured using a Chroma Meter (Miniscan XE, Hunter Lab), and the meter was calibrated using a white tile. A 100 ml beaker containing 40 ml of tea infusions was placed on a standard white plate. Three measurements of L*, a*, and b* values representing lightness, red to green color dimension, and yellow to blue color dimension, respectively, were taken for each sample by placing the Chroma meter directly on top of the tea infusion. Hue angle was calculated as Hue = tan^−1^(b*/a*).

### 2.7 Antioxidant capacity by 1,1‐Diphenyl‐2‐picrylhydrazyl radical assay

Free radical scavenging ability of tea infusion was measured by 1,1‐Diphenyl‐2‐picrylhydrazyl (DPPH) radical scavenging method with slight modifications (Wu et al., 2011). DPPH reagent was prepared in ethanol (100 μmol/L), and 50 μl was added to 10 ml of tea infusion. The mixture was placed in the dark for 30 min. The scavenging activity was quantified by measuring the absorbance at 517 nm using the spectrophotometer described above. The control was prepared by mixing deionized water with DPPH reagent instead of ethanol. A mixture of 50 μl of ethanol and 10 ml of tea infusion was served as the blank. Results were expressed as the percentage scavenging of DPPH radical calculated using the following equation:

$$\text{DPPH radical scavenging activity}=\frac{{\text{Abs}}_{0}‐\left({\text{Abs}}_{1}‐{\text{Abs}}_{2}\right)}{{\text{Abs}}_{0}}\times 100\%$$

where Abs_0_ is the absorbance of the control, Abs_1_ is the absorbance of the sample, and Abs_2_ is the blank.

All chemicals used for analysis were analytical reagent grade obtained from VWR International.

### 2.8 Statistical analysis

All experiments and sample analysis were carried out in triplicate, and the average values were calculated. Results were expressed as the mean ± standard deviation (mean ± *SD*, *n* = 3) for each analysis. Student's *t* test was performed using the JMP Pro 13 (SAS Institute, Inc.). A *p *< .05 was considered as significant.

## 3 RESULTS AND DISCUSSION

3

### 3.1 Brewing conditions effect on chemical compounds in tea infusion

Some nonvolatile compounds, such as soluble sugars, free amino acids, polyphenols, and caffeine are responsible for taste in black tea (Alasalvar et al., 2012). Table 1 shows the concentration (μg/ml) of soluble sugars, polyphenols, caffeine, and total amino acids in tea infusions, and their total content for each extraction per unit weight of tea powder used (mg/cup.g). One way to evaluate the extraction efficiency is to measure the concentration of each compound in tea infusions. For example, soluble sugars concentration in tea infusion using 1.0 g of 0.60 mm tea powders decreased from 148.9 to 91.7 μg/ml when the total amount of water used for extraction increased from 118 to 236 ml (Table 1). When using 1.0 g of tea powders but particle size decreased from 0.60 to 0.30 mm, the concentration of soluble sugars increased from 148.9 to 227.5, 110.9 to 176.9, and 91.7 to 136.8 μg/ml for 118, 177, and 236 ml water, respectively. Farakte, Yadav, Joshi, Patwadhan, and Singh (2016) also found the smaller the particle size, the faster the infusion rate for black tea. The findings also support the results from the current study that using the smallest tea powder will achieve the highest extraction efficiency (Table 1). When the amount of tea powders used for brewing increased to 1.5 or 2.0 g, effects of particle size and volume of water used for extraction followed a similar trend as using 1.0 g tea powders. Concentration of soluble sugars increased from 149.8 to 178.3 μg/ml when the amount of tea powder increased from 1.0 to 1.5 g (0.60 mm tea powder and 118 ml water for brewing). The extraction concentration was the highest (412.3 μg/ml) when using the highest amount of finest tea powder and the smallest amount of water (118 ml).

**Table 1:** Extraction of chemical compounds in tea infusion using a single‐serve coffee maker

Powder weight (g)	Particle size mm)	Water volume (ml)	Concentrations (μg/ml)				Total extraction (mg/cup.g)				
			Soluble sugars	Polyphenols	Caffeine	Amino acids		Soluble sugars	Polyphenols	Caffeine	Amino acids
1.0	0.60	118	148.9 ± 1.7^L^	96.8 ± 1.4^L^	88.3 ± 2.5^K^	65.1 ± 0.5^K^		17.6 ± 0.2	11.5 ± 0.2	10.4 ± 0.2	7.7 ± 0.1
1.0	0.60	177	110.9 ± 1.5^P^	69.1 ± 0.9^N^	64.6 ± 1.2^M^	46.1 ± 0.6^N^		19.6 ± 0.3	12.2 ± 0.2	11.3 ± 0.2	8.2 ± 0.1
1.0	0.60	236	91.7 ± 1.9^Q^	60.2 ± 1.2^O^	62.4 ± 1.6^N^	35.1 ± 0.7^O^		21.6 ± 0.4	14.0 ± 0.3	14.7 ± 0.3	8.3 ± 0.2
1.0	0.30	118	227.5 ± 2.3^G^	129.2 ± 1.3^H^	110.7 ± 2.2^G^	91.6 ± 1.3^I^		26.8 ± 0.3	15.1 ± 0.4	13.1 ± 0.2	10.8 ± 0.2
1.0	0.30	177	176.9 ± 3.2^I^	105.6 ± 0.7^K^	89.1 ± 2.6^K^	63.5 ± 1.3^Kl^		31.3 ± 0.6	18.7 ± 0.3	15.8 ± 0.3	11.2 ± 0.2
1.0	0.30	236	136.8 ± 2.3^N^	88.1 ± 1.6^M^	68.9 ± 1.2^L^	51.6 ± 1.6^M^		32.3 ± 0.3	20.8 ± 0.2	16.3 ± 0.3	12.2 ± 0.4
1.5	0.60	118	178.3 ± 3.1^J^	122.8 ± 2.9^I^	115.1 ± 2.1^F^	99.4 ± 2.5^G^		14.0 ± 0.4	9.7 ± 0.2	9.0 ± 0.2	7.8 ± 0.3
1.5	0.60	177	141.8 ± 2.8^M^	116.6 ± 1.5^J^	105.6 ± 2.3^H^	82.7 ± 1.3^J^		16.7 ± 0.5	13.7 ± 0.3	12.5 ± 0.3	9.8 ± 0.2
1.5	0.60	236	119.2 ± 2.7 ^O^	96.8 ± 1.7^L^	92.8 ± 1.7^J^	62.7 ± 1.2^L^		18.7 ± 0.6	15.1 ± 0.2	14.6 ± 0.3	9.9 ± 0.3
1.5	0.30	118	348.9 ± 6.1^B^	206.3 ± 2.1^B^	154.9 ± 3.3^B^	160.1 ± 3.2^B^		27.4 ± 0.7	16.2 ± 0.1	12.2 ± 0.3	12.6 ± 0.4
1.5	0.30	177	235.1 ± 4.7^E^	157.0 ± 1.7^F^	130.7 ± 2.4^D^	98.6 ± 1.8^G^		27.7 ± 0.8	18.4 ± 0.1	15.4 ± 0.3	11.6 ± 0.3
1.5	0.30	236	168.1 ± 2.3^K^	140.8 ± 1.6^G^	100.9 ± 1.8^I^	81.6 ± 1.4^J^		26.4 ± 0.5	22.3 ± 0.2	15.9 ± 0.2	12.8 ± 0.3
2.0	0.60	118	229.1 ± 3.5^F^	159.5 ± 0.9 ^E^	151.8 ± 3.6^C^	149.4 ± 1.8^C^		13.5 ± 0.4	9.4 ± 0.1	9.0 ± 0.2	8.8 ± 0.2
2.0	0.60	177	203.3 ± 3.7^H^	142.6 ± 1.7^G^	127.1 ± 2.4^E^	111.2 ± 3.1^F^		18.0 ± 0.7	12.6 ± 0.2	11.2 ± 0.2	9.8 ± 0.5
2.0	0.60	236	192.3 ± 3.1^I^	130.3 ± 1.3^H^	115.6 ± 2.6^F^	95.5 ± 1.9^H^		22.7 ± 0.7	15.5 ± 0.3	13.6 ± 0.2	11.3 ± 0.4
2.0	0.30	118	412.3 ± 6.1^A^	251.6 ± 2.4^A^	208.9 ± 4.1^A^	205.3 ± 3.6^A^		24.3 ± 0.7	14.8 ± 0.2	12.3 ± 0.2	12.1 ± 0.4
2.0	0.30	177	327.4 ± 5.1^C^	191.2 ± 1.2^C^	156.6 ± 3.4^B^	141.5 ± 1.4^D^		29.0 ± 0.9	16.9 ± 0.2	13.9 ± 0.3	12.5 ± 0.2
2.0	0.30	236	256.4 ± 4.6^D^	164.5 ± 2.7^D^	131.5 ± 2.3^D^	115.1 ± 2.1^E^		30.3 ± 1.1	19.4 ± 0.3	15.5 ± 0.3	13.6 ± 0.5

Note

Values with the same letter in the same column are not significant different (*P* ≥ .05).

Another way to evaluate the extraction efficiency is to measure the total amount of each compound extracted during each brewing for each unit weight of tea powders. When the amount of water used for brewing increased from 118 to 236 ml, the total amount of soluble sugars in each cup of tea infusions increased from 17.6 to 21.6 mg/cup when using 1.0 g of 0.60 mm tea powders. At the same extraction condition as discussed above but using finer tea powders (0.30 mm vs. 0.60 mm), soluble sugars in each cup of tea infusions increased from 17.6, 19.6, and 21.6 to 26.8, 31.3, and 32.3 mg/cup for 118, 177, and 236 ml water, respectively. The extraction efficiency was the highest (32.3 mg/cup.g tea) for a unit amount of tea powders was at the smallest particle size (0.30 mm) and with the highest amount of water (236 ml). Further increase the amount of tea powders used for extraction, the total amount of soluble sugars in each cup (mg/cup) increased (data not show); however, the total amount of soluble sugars in each cup per unit weight of tea powders actually decreased (32.3–26.4 for 1.0 and 1.5 g tea powders, respectively). As discussed earlier, when increasing the amount of tea powders from 1.0 to 2.0 g, soluble sugars in each cup increased from 227.5 to 413.3 μg/ml; however, the total amount of soluble sugar in each cup per unit weight of tea powder actually decreased (Table 1).

For tea infusion preparation, if the purpose is to have the highest concentration of soluble sugars, the extraction condition should be using the highest amount of the finest tea powder with the smallest amount of water. However, if the purpose is to extract the highest amount of soluble sugars from tea powders, the extraction condition should be using the smallest amount of the finest tea powders with the highest amount of water. In addition, soluble sugars can contribute to a sweet taste for tea infusion (Sai, Chaturvedula, & Prakash, 2011), and a high content of soluble sugars also has a conciliatory effect on the bitterness and astringency of tea (Lin et al., 2014). In general, relative high content of soluble sugars is good for tea drinks.

Caffeine is an alkaloid compound found in various plants such as tea leaves, coffee, and cocoa. It can stimulate the central nervous system and improve mood (Ruxton, 2008; Verdiani et al., 2018). As showed in Table 1, the effect of water volume, tea powder particle size, and the amount of tea powder used on the infusion followed the same trend as soluble sugars (Table 1), and the highest concentration was 208.9 μg/ml (2.0 g of 0.30 mm tea powders with 118 ml water), and the highest total extraction efficacy was 16.3 mg/g.cup (1.0 g of 0.30 mm tea powders with 236 ml water).

The concentration of amino acids in tea infusion made by 1.0 g of 0.60 mm tea powders decreased with increasing the amount of water used, followed the same trend as soluble sugars (Table 1). When particle size was reduced to 0.30 mm, the concentration of amino acids in tea infusion increased. When the amount of tea powders increased to 1.5 or 2.0 g, effects of particle size and volume of water followed a similar trend as 1.0 g tea powders. Extraction efficacy was the highest (205.3 μg/ml) when using 2.0 g of smaller particle size (0.30 mm) tea powders and with the highest amount of water (236 ml). Amino acids contribute to the fresh taste of tea, and it is also associated with the enhancement of relaxation (Vuong, Bowyer, & Roach, 2011). Many studies have found amino acids content are highly correlated with the quality and the taste of green and black tea infusions (Alcazar et al., 2007; Ding, Yu, & Mou, 2002; Kaneko, Kumazawa, Masuda, Henze, & Hofmann, 2006).

The highest concentration of polyphenols in tea infusion (251.6 μg/ml) was achieved by using 2.0 g of 0.30 mm tea powders with 118 ml of water, whereas the highest extraction efficiency (22.3 mg/cup.g) was observed at using 1.5 g of 0.30 mm tea powders with the highest amount of water (236 ml). Tea drinks in general contain multiple polyphenols, for example, catechins, theaflavins, tannins, and flavonoids. These chemical compounds affect the flavor and mouthfeel and are speculated to provide potential health benefits (Riegsecker, Wiczynski, Kaplan, & Ahmed, 2013; Yang & Liu., 2013).

The average effects of tea brewing parameters on tea infusion chemical composition were summarized in Table 2. The maximum concentration of the four compounds were obtained using 2.0 g of tea powders, which were 270.1, 906.3, 173.2, and 136.3 μg/ml for soluble sugars, caffeine, polyphenols, and amino acid, respectively. As for the amount of water for brewing, the less the amount of water used, the greater the concentration (Table 2). For tea powder particle size effect, the maximum concentration was obtained by using 0.30 mm (smallest powder size) tea powders and achieved 1.25–1.58 folder higher concentration than 0.60 mm tea powders. These results are expected; however, the total amount of chemical compounds extracted into the tea infusions during a short brewing time is also affected by the solubility of each chemical component in water. Soluble sugars and caffeine are highly soluble in hot water (Musilova & Kubickova, 2018). Hence, the total amount of soluble sugars and caffeine in each cup for each unit weight of tea powders is using the smallest amount of smallest tea powders with highest amount of water. Amino acid and polyphenols are less soluble in water than the other two components discussed above, and hence, higher amount of tea powders are needed to reach the highest total extraction per unit weight of tea powders.

**Table 2:** Tea infusion composition as affected by the amount of tea powder, water volume, and particle size

Item	Quantity	Soluble sugars (µg/ml)	Caffeine (µg/ml)	Polyphenols (µg/ml)	Amino acids (µg/ml)
Amount of tea (g)	1	148.8	492.2	91.5	58.8
	1.5	198.6	711.6	140.0	97.5
	2	270.1	906.3	173.2	136.3
Amount of water (ml)	118	256.4	843.6	160.9	128.5
	177	205.3	684.9	130.3	90.6
	236	160.8	581.6	112.0	73.6
Particle size (mm)	0.60	160.6	625.7	110.5	83.0
	0.30	254.4	781.0	159.3	112.1

For the traditional brewing method, 5 g tea leaves were extracted using 110 ml of hot water during each brew for up to seven times. It is difficult to compare the two brewing methods in this study. The single‐serve coffee maker method was one‐time extraction using tea powders whereas the traditional method using large amount of tea leaves (5.0 g) and can brew 7 times. The soluble sugars concentration per unit weight of tea was 78.8 μg/ml.g from the first brew and gradually increased with the number of brew and reached the maximum concentration of 102.7 μg/ml.g during the third brew and then decreased rapidly with further brew (Table 3). The reason for the maximum concentration was achieved during the third brew maybe due to the reason that dried tea leaves were used and require time for leaves to rehydrate. As discussed before, soluble sugars are soluble in water and hence can be quickly extracted from tea leaves (during the first 3 brews) and concentration in the later brews decreased quickly. Caffeine concentration followed a similar trend as soluble sugars and reached the highest concentration (74.6 μg/ml.g) at the second brew and decreased rapidly from the fourth brew. This is also because caffeine is highly soluble in hot water. For polyphenols and amino acids, highest concentration was also reached at the third brew (55.9 and 50.2 μg/ml.g, respectively). However, the concentration decreased at a much slower rate than soluble sugars and caffeine during the fourth and late brews. This may due to the low solubility in water and hence will be extracted gradually. Although it is difficult to compare the chemical compound concentration in tea infusions by two different methods, we can at least compare the highest (the third) brew from the traditional method with the single‐serve coffee maker method using 1.0 g of 0.30 mm of tea powder with 118 ml of water. By dividing the total extraction values (mg/cup.g) from Table 1 by 118 ml, the concentration of soluble sugars, polyphenols, caffeine, and amino acids is 227.1, 128.0, 110.2, and 91.5 μg/ml.g, respectively, and these values are much higher than 102.7, 59.9, 68.6, and 50.2 μg/ml.g from the third brew of the traditional method. This may indicate the single‐serve coffee maker method may be a convenient method to brew high quality of tea infusions.

**Table 3:** Chemical compounds concentration in tea infusion brewed using a traditional method

Brew	Concentrations (μg/ml)				Concentrations (μg/ml.g)			
	Soluble sugars	Polyphenols	Caffeine	Amino acids	Soluble sugars	Polyphenols	Caffeine	Amino acids
1	393.9 ± 7.2^C^	199.3 ± 4.1^D^	284.2 ± 5.1^C^	215.2 ± 3.9^B^	78.8 ± 1.4	39.9 ± 0.8	56.8 ± 1.0	43.0 ± 0.8
2	425.8 ± 7.9^B^	237.2 ± 3.6^B^	373.2 ± 6.2^A^	209.5 ± 3.4^C^	85.2 ± 1.6	47.4 ± 0.7	74.6 ± 1.2	41.9 ± 0.7
3	513.5 ± 9.3^A^	299.3 ± 5.7^A^	342.8 ± 5.9^B^	250.8 ± 4.7^A^	102.7 ± 1.8	59.9 ± 1.1	68.6 ± 1.2	50.2 ± 0.9
4	272.5 ± 6.1^D^	212.0 ± 3.9^C^	188.3 ± 2.8^D^	155.9 ± 2.8^D^	54.5 ± 1.2	42.4 ± 0.8	37.7 ± 0.6	31.2 ± 0.6
5	257.8 ± 5.8^E^	195.1 ± 2.7^E^	152.0 ± 2.7^E^	152.1 ± 2.3^E^	51.6 ± 1.2	39.0 ± 0.5	30.4 ± 0.5	30.4 ± 0.5
6	180.7 ± 3.5^F^	149.3 ± 2.3^F^	88.9 ± 1.6^F^	124.6 ± 1.7^F^	36.1 ± 0.7	29.9 ± 0.4	17.8 ± 0.3	24.9 ± 1.7
7	142.2 ± 2.5^G^	136.3 ± 2.4^G^	51.9 ± 1.1^G^	118.1 ± 1.2^G^	28.4 ± 0.5	27.2 ± 0.5	10.4 ± 0.2	23.6 ± 0.3
Average	312.4	204.1	211.6	175.9	62.5	40.8	42.3	35.2

Note

Value with the same letter in the same column are not significant different (*P* ≥ .05).

### 3.2 Brewing conditions effect on antioxidant activities

DPPH is a free radical featuring an intense purple color in solution and has a strong absorption band at the wave length of 515–520 nm (Rodrigues, da Silva, dos Santos, Zielinski, & Haminiuk, 2015). In general, a product with high free radicals scavenging capacity will achieve strong DPPH reduction. Thus, by measuring the antioxidant capacity of tea infusion, it may reflect the possible health benefits through radical scavenging effect (Li et al., 2013; Sharpe et al., 2016).

Table 4 shows the results of antioxidant capacity activity of the tea infusions. Tea infusions prepared using 1.0 g of 0.6 mm tea powders, DPPH radical scavenging capacity decreased slightly as the amount of water used for brewing increased from 118 to 236 ml. When tea particle size reduced to 0.30 mm, DPPH radical scavenging capacity increased from 45.1% to 81.6%, 42.6% to 62.5%, and 38.5% to 57.4% at 118, 177, and 236 ml, respectively. This indicates tea infusion has strong antioxidant capacity when tea powder particle size reduced and make the extraction of tea compounds like polyphenols quicker (Table 1). Effects of tea powder size and amount of brewing water on DPPH radical scavenging capacity followed a similar trend as other chemical compounds (Table 1) when the amount of tea used for brewing increased to 1.5 or 2.0 g. However, radical scavenging capacity for tea infusions made with 1.5 or 2.0 g of 0.3 mm tea powders was all higher than 85% and not significantly affected by the amount of tea powders and water volume. This may be due to the high radical scavenging capacity and most of the DPPH radical already been consumed (Table 4).

**Table 4:** Radical scavenging capability of tea infusion

Tea powder particle size (mm)	Powder weight (g)	Water volume (ml)	Scavenging rate (%)
0.6	1.0	118	45.1 ± 1.1^K^
0.6	1.0	177	42.6 ± 1.3 ^L^
0.6	1.0	236	38.5 ± 2.7^M^
0.6	1.5	118	83.9 ± 2.1^E^
0.6	1.5	177	81.6 ± 1.3^F^
0.6	1.5	236	79.2 ± 2.2^G^
0.6	2.0	118	89.4 ± 0.8^D^
0.6	2.0	177	80.7 ± 1.2^F^
0.6	2.0	236	72.3 ± 1.3^H^
0.3	1.0	118	81.6 ± 1.2^F^
0.3	1.0	177	62.5 ± 0.4^I^
0.3	1.0	236	57.4 ± 1.1^J^
0.3	1.5	118	91.7 ± 2.3^BC^
0.3	1.5	177	88.6 ± 1.2^D^
0.3	1.5	236	85.1 ± 2.7^E^
0.3	2.0	118	94.7 ± 1.3^A^
0.3	2.0	177	92.9 ± 1.5^B^
0.3	2.0	236	91.4 ± 0.8^C^

Note

Values with the same letter in the same column are not significant different (*p* ≥ .05).

A higher DPPH radical scavenging capacity indicates stronger antioxidant capacity of the tea infusion. Research shows that tea polyphenols are major antioxidants and can effectively remove free radicals (Wang, Zhao, Andrae‐Marobela, Okatch, & Xiao, 2013). Tea infusion brewed using more tea powder with smaller size particle can extract more polyphenols (Table 2). Research indicated that polyphenols, flavonoids, and epigallocatechin gallate showed a significant correlation with the antioxidant activity of tea (Fernando & Soysa, 2015). Current study also found tea infusions prepared by a single‐serve coffee maker have strong free radical scavenging capacity (Table 4), indicating a benefit of tea drinking.

### 3.3 Effect on Color

Color is an important quality indicator for a tea infusion. As shown in Table 5, L* value of tea infusion made by 1.0 g of 0.60 mm tea powders, increased from 10.9 to 17.7 (indicating lighter color) as water increased from 118 to 236 ml and indicates the tea infusion become less concentrate when water volume was increased. When tea powder particle size reduced to 0.30 mm, the *L value decreased from 10.9 to 4.1, 12.2 to 6.1, and 17.7 to 11.6 for 118, 177, and 236 ml water, respectively. This indicated tea infusion became more concentrated when using smaller tea powder. When the amount of tea powder used increased to 1.5 or 2.0 g, effects of tea powder size and the amount of brewing water used followed a similar trend as using 1.0 g tea powder. Tea infusion made with 2.0 g of 0.30 mm tea powders and 118 ml of water had the lowest L* value indicating having the highest tea infusion concentration. When tea infusion was prepared by using 1.0 g of 0.60 mm tea powder and 236 ml water, tea infusion had the highest L* value indicating it had the lowest concentration. These also support the findings on tea infusion chemical compound concentration presented in Table 1.

**Table 5:** Color property of tea infusion

Powder weight (g)	Particle size (mm)	Water volume (ml)	*L* ^*^	a*	*b* ^*^	Hue angle
1.0	0.60	118	10.9 ± 0.1^DE^	20.7 ± 0.4^G^	12.7 ± 0.2^BC^	31.5 ± 1.8^E^
1.0	0.60	177	12.2 ± 0.07^C^	17.4 ± 0.3^H^	13.2 ± 0.1^B^	37.2 ± 1.2^D^
1.0	0.60	236	17.7 ± 0.09^A^	13.8 ± 0.4^J^	15.1 ± 0.3^A^	47.6 ± 0.6^A^
1.0	0.30	118	4.1 ± 0.05^I^	22.5 ± 0.2^F^	9.4 ± 0.07^H^	22.9 ± 0.6^J^
1.0	0.30	177	6.1 ± 0.07^GH^	20.7 ± 0.3^G^	10.3 ± 0.1^FG^	26.4 ± 1.2^H^
1.0	0.30	236	11.6 ± 0.1^CD^	15.0 ± 0.1^I^	12.4 ± 0.1^C^	39.5 ± 0.7^C^
1.5	0.60	118	6.6 ± 0.07^G^	23.6 ± 0.1^EF^	9.5 ± 0.04^H^	21.8 ± 1.9^K^
1.5	0.60	177	10.2 ± 0.09^E^	22.1 ± 0.4^F^	11.5 ± 0.2^D^	27.5 ± 1.3^G^
1.5	0.60	236	14.3 ± 0.06^B^	15.3 ± 0.2^I^	14.6 ± 0.2^A^	43.5 ± 2.1^B^
1.5	0.30	118	2.3 ± 0.02^JK^	26.1 ± 0.09 ^BC^	7.1 ± 0.08^J^	14.9 ± 1.3^N^
1.5	0.30	177	4.4 ± 0.03^I^	24.2 ± 0.1^DE^	8.6 ± 0.06^I^	19.5 ± 0.8^L^
1.5	0.30	236	9.3 ± 0.04^F^	18.6 ± 0.3^H^	10.8 ± 0.2^EF^	30.1 ± 1.9^F^
2.0	0.60	118	3.2 ± 0.05^J^	26.9 ± 0.1^B^	8.2 ± 0.05^I^	17.2 ± 2.2^M^
2.0	0.60	177	5.3 ± 0.02^HI^	24.6 ± 0.4^DE^	9.8 ± 0.1^GH^	21.8 ± 1.4^K^
2.0	0.60	236	8.9 ± 0.09^F^	18.3 ± 0.5^H^	11.3 ± 0.1^DE^	31.5 ± 0.8^E^
2.0	0.30	118	0.7 ± 0.02^L^	28.3 ± 0.1^A^	6.1 ± 0.1^K^	12.0 ± 2.5^O^
2.0	0.30	177	1.5 ± 0.08^Kl^	25.3 ± 0.1^CD^	7.5 ± 0.2^J^	16.6 ± 0.7^M^
2.0	0.30	236	6.8 ± 0.07^G^	20.4 ± 0.3^G^	9.5 ± 0.1^H^	24.6 ± 1.1^I^

Note

Values with the same letter in the same column are not significant different (*p* ≥ .05).

Effect of tea particle size and the amount of tea and water used on Hue angle followed a similar trend as L* value. When Hue angle is between 10 and 60, the higher the Hue value, the more orange of the color, while the lower the Hue angle value, the darker brown or reddish the color. The hue angle of tea infusion, made by using 1.0 g of 0.60 mm tea powder, increased as the amount of water for brewing increased, but Hue angle value decreased as tea particle decreased. When increasing the amount of tea powder used from 1.0 to 1.5 g or 2.0 g, the hue angle value further decreased. Tea infusion made with 2.0 g of 0.3 mm tea powders using 118 ml water had the highest chemical compound concentration (Table 1) and the darkest brown color (lowest hue angle value at 12.0).

## 4 CONCLUSION

Study showed that the chemical composition of tea infusions brewed with a single‐serve coffee maker could be affected by brewing conditions, such as the quantity of tea, the tea powder particle size, and water volume. Using the highest amount of tea with the smallest particle size and the smallest amount of water could get tea infusion with the highest concentration of chemical compounds. However, for the extraction efficiency, the best condition for soluble sugars and caffeine were obtained by using 1.0 g of 0.3 mm tea powder with 238 ml water, for polyphenols was using1.5 g of 0.3 mm tea powders, while amino acids was 2.0 g of 0.3 mm tea powders with 238 ml water, respectively.

The average contents of soluble sugars, tea polyphenols, caffeine, and amino acid in infusion brewed by the traditional method were 312.4, 204.1, 211.6, and 175.9 μg/ml, respectively. However, 5.0 g of tea leaves were used for traditional brewing and much higher than the 1.0–2.0 g of tea powders used for the single‐server brewing studies. The calculated chemical compound concentrations in tea infusions made with 1 g of 0.30 mm tea powder with 118 ml water per unit weight of tea were 227.5, 129.2, 110.0, and 91.6 μg/ml.g for soluble sugars, polyphenols, caffeine, and amino acids, respectively, and higher than the highest concentration of 102.7, 55.9, 68.6, and 50.2 μg/ml.g from the third brew of traditional method. Tea infusions prepared by the single‐serve coffee maker have strong DPPH free radical scavenging capacity, and this may be due to the high polyphenol content in the tea infusion and hence stronger antioxidant capacity. Tea color was also affected by the concentration of the chemical compounds, and tea infusion with the highest chemical compound concentration also had the lowest L* value indication the darkest color.

Results obtained from this study demonstrated that the single‐serve coffee maker can be a convenience tea brewing method and tea infusion quality can be optimized based on consumer preference. For example, if consumers prefer tea infusion with the highest tea chemical compound concentration, the highest amount of smallest size tea powders and smallest amount of water should be used. If consumers want to extract the most of chemical components from tea powders for a cup of tea, the smallest of tea powders and the highest amount of water should be used.

## CONFLICT OF INTEREST

The authors declare no conflict of interest.

## AUTHOR CONTRIBUTIONS

Chunhua Ma collected test data and drafted the manuscript, and Yen‐Con Hung designed the study and revised the manuscript.

## ETHICAL APPROVAL

Ethical Review: This study does not involve any human or animal testing.
